# Large Buccal Space Lipoma Excised Through an Intraoral Approach

**DOI:** 10.7759/cureus.70475

**Published:** 2024-09-29

**Authors:** Konstantinos Katoumas, Maria Kouri, Dimitrios Anterriotis, Maria Georgaki, Nikolaos G Nikitakis

**Affiliations:** 1 Department of Oral and Maxillofacial Surgery, School of Dentistry, National and Kapodistrian University of Athens, Athens, GRC; 2 Department of Oral Medicine and Pathology and Hospital Dentistry, School of Dentistry, National and Kapodistrian University of Athens, Athens, GRC; 3 Department of Oral and Maxillofacial Surgery, General Hospital of Athens «G. Gennimatas», Athens, GRC

**Keywords:** buccal fat pad, buccal space, buccal tumor, intraoral approach, lipoma

## Abstract

Lipomas are benign tumors of adipose tissue. They represent the most common mesenchymal neoplasm but are relatively rare in the oral and maxillofacial regions. The purpose of this study is to present an unusual case of a large lipoma of the buccal space and its excision by an intraoral approach. A 38-year-old male patient presented with an otherwise asymptomatic swelling of the right cheek that had first been noticed four years earlier and had subsequently exhibited gradual, continuous enlargement with stable dimensions over the last year. The patient was obese but otherwise healthy. Examination revealed a movable, well-circumscribed, non-fluctuant, soft-elastic large swelling of the right buccal and parotid-masseteric region with normal overlying skin and no bruit. Ultrasound and MRI findings, as well as fine needle aspiration, were suggestive of lipoma. The lesion was excised under general anesthesia through an intraoral approach. The tumor measured 7.0 cm × 5.3 cm × 1.6 cm and was separated from the surrounding tissues by a thin capsule. Histopathologic examination rendered a final diagnosis of lipoma. No signs of recurrence were noted. Although lipomas are the most common mesenchymal neoplasm, they do not usually occur in the oral and maxillofacial region, especially as large lesions are located in the buccal space, and may pose diagnostic and therapeutic challenges.

## Introduction

Lipomas are benign soft tissue neoplasms of adipose tissue. Many lipomas remain unrecorded and receive little interest in the literature since most of them grow insidiously and may become stabilized, causing few symptoms other than the presence of a localized mass [1]. Most lipomas come to the attention only when they grow larger, causing a cosmetic problem or interfering with function due to their anatomic location.

As a matter of fact, the number of reported cases of lipomas is probably much lower than the actual incidence [1], considering that lipomas represent by far the most common mesenchymal neoplasm [1,2]. Lipomatous tumors, in total, have an estimated annual incidence of 1 per 1,000 inhabitants, with benign tumors outnumbering their malignant counterparts by 100 to 1 [3,4]. However, only 1% to 4% of all lipomas are located in the oral and maxillofacial regions [5-7].

The aim of the study is to present a rare case of a large lipoma of the right buccal space, which was excised intraorally.

## Case presentation

A 38-year-old male patient visited the Oral and Maxillofacial Surgery Clinic with a chief complaint of swelling of the right cheek. The patient reported that the swelling had first been noticed four years earlier and had subsequently exhibited gradual continuous enlargement with stable dimensions over the last year; it was otherwise asymptomatic. Despite being obese, the patient did not have any other health issues or a family history of lipomas, nor did they report any specific predisposing events.

**Figure 1:**
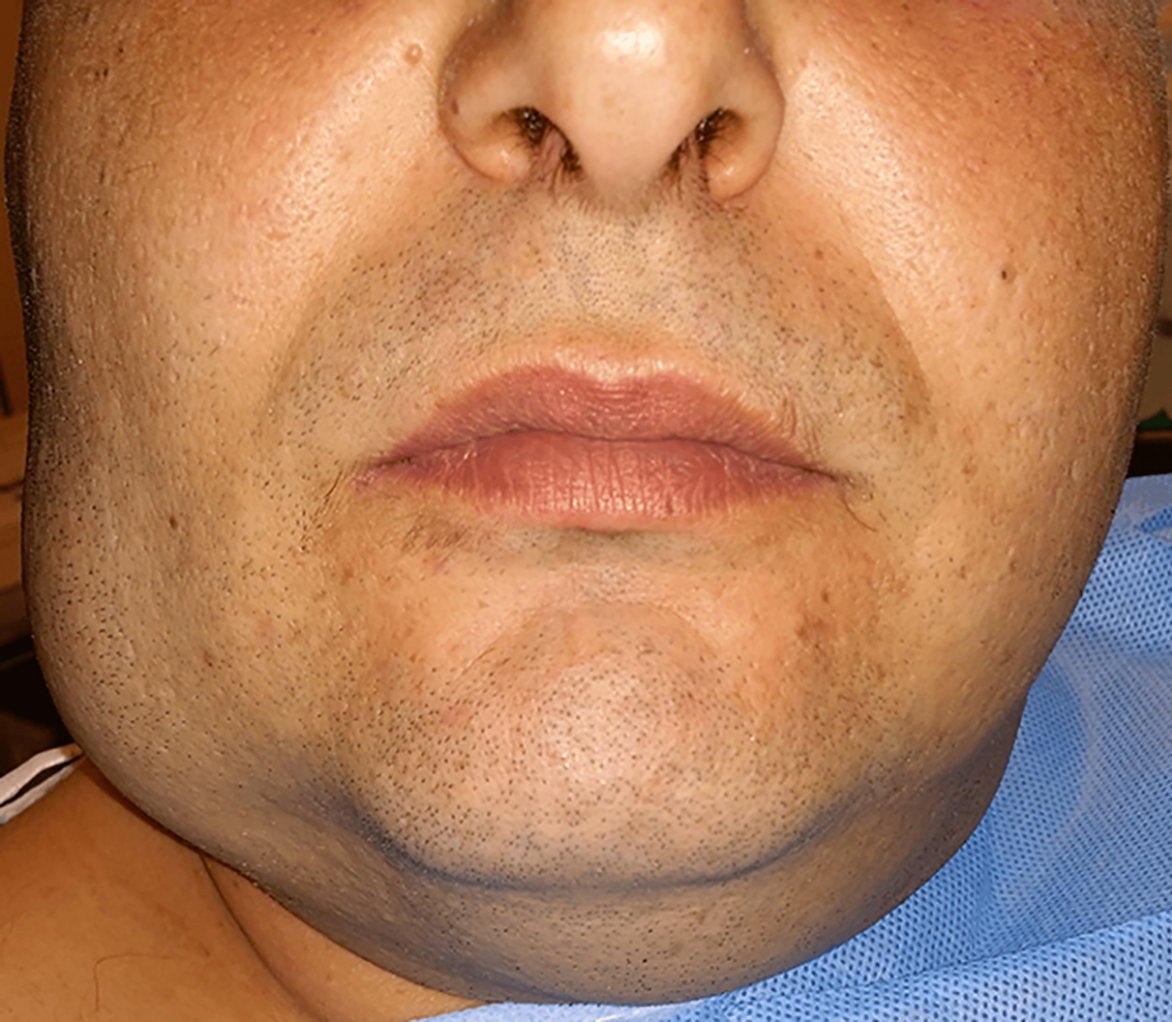
Large swelling of the right buccal and parotid-masseteric region

**Figure 2:**
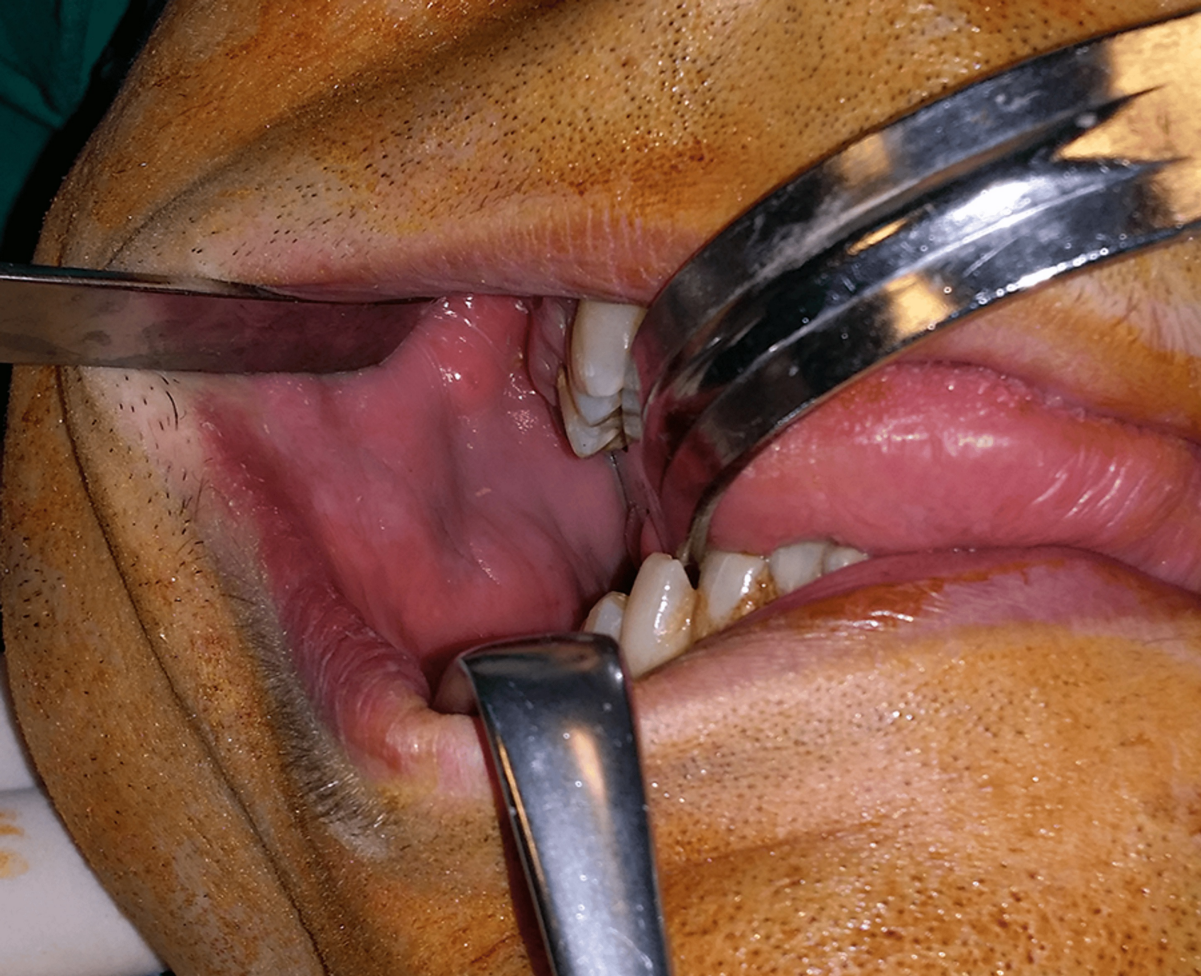
Small, non-fluctuant swelling in the buccal mucosa

A meticulous clinical oral and maxillofacial examination was performed. On extraoral examination, a large swelling of the right buccal and parotid-masseteric region was apparent (Figure 1). A well-circumscribed soft-elastic in consistency and non-fluctuant, movable tumor with normal overlying skin and no bruit present could be easily palpated. Intraoral examination revealed a smaller, non-fluctuant swelling in the buccal mucosa (Figure 2). Except for poor oral hygiene, no other oral findings were noticed.

**Figure 3:**
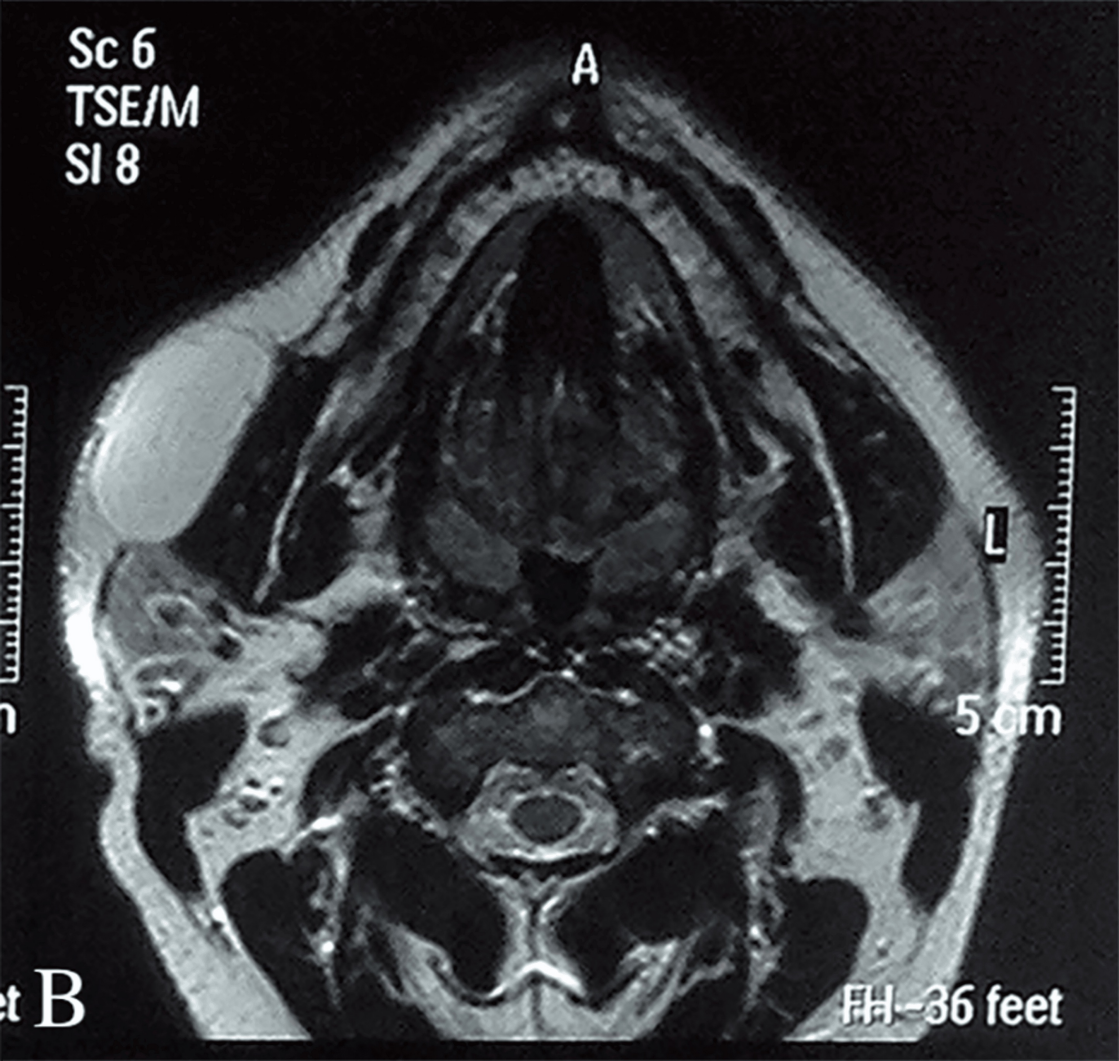
MRI scan revealed a large fatty mass laterally of the buccinator muscle

A provisional clinical diagnosis of mesenchymal or salivary gland tumor was made. An ultrasound examination of the swelling was performed, revealing a lesion with well-defined borders, no vascularity of the lesion in color Doppler, and characteristics suggestive of lipoma. Due to the tumor size, the patient was referred for an MRI scan, which revealed a large fatty mass laterally in the buccinator muscle (Figure 3). The lesion was in close proximity to the masseter muscle, had well-defined borders with no signs of infiltration, and showed high signal intensity on both T1- and T2-weighted images and low signal intensity in fat-suppressed sequences. The administration of IV contrast agent did not show any enhancement. MRI features were consistent with a lipoma. Before definitive treatment, the patient was also referred for a fine needle aspiration and cytology examination that was compatible with lipoma.

**Figure 4:**
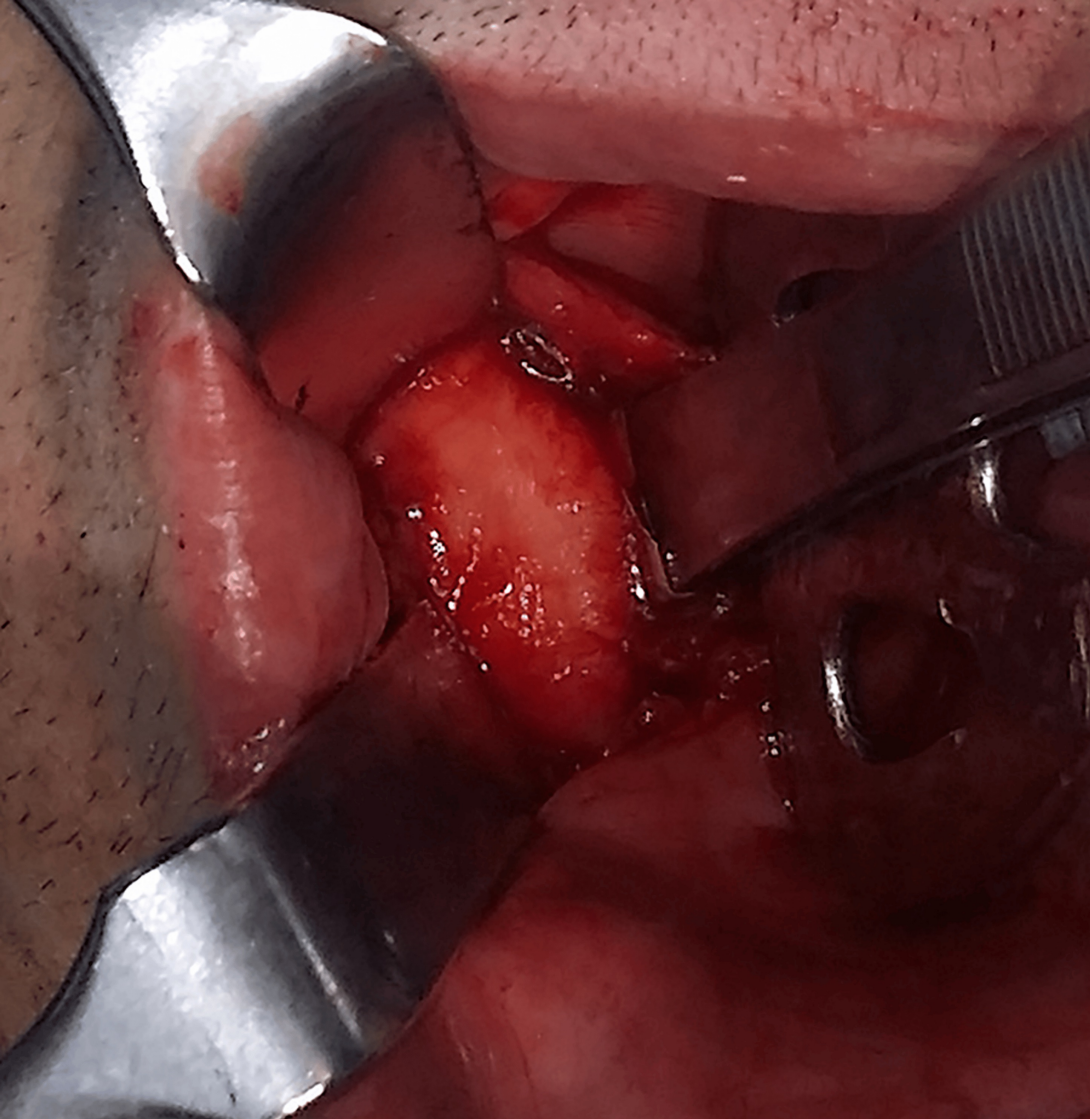
Aspect of the tumor through the intraoral incision

The excision of the lesion was performed under general anesthesia through an intraoral approach (Figure 4). After disinfection of the area and recognition of the parotid papilla, a local anesthetic solution with vasoconstrictor was administered. The buccal mucosa was incised with a no. 15 blade from posterior to anterior below the Stenson duct, and then the buccinator muscle was incised in a similar fashion with a Colorado radiofrequency diathermy. Upon tumor recognition, its capsule was meticulously separated from the surrounding tissues, taking into consideration the buccal branches of the facial nerve. The masseter muscle, surrounding vessels, and parotid duct were identified and protected as well. After tumor excision, thorough hemostasis and rinse with povidone-iodine, hydrogen peroxide, and then copious saline were performed, and the buccinator muscle and buccal mucosa were sutured by planes with resorbable 3-0 sutures.

**Figure 5:**
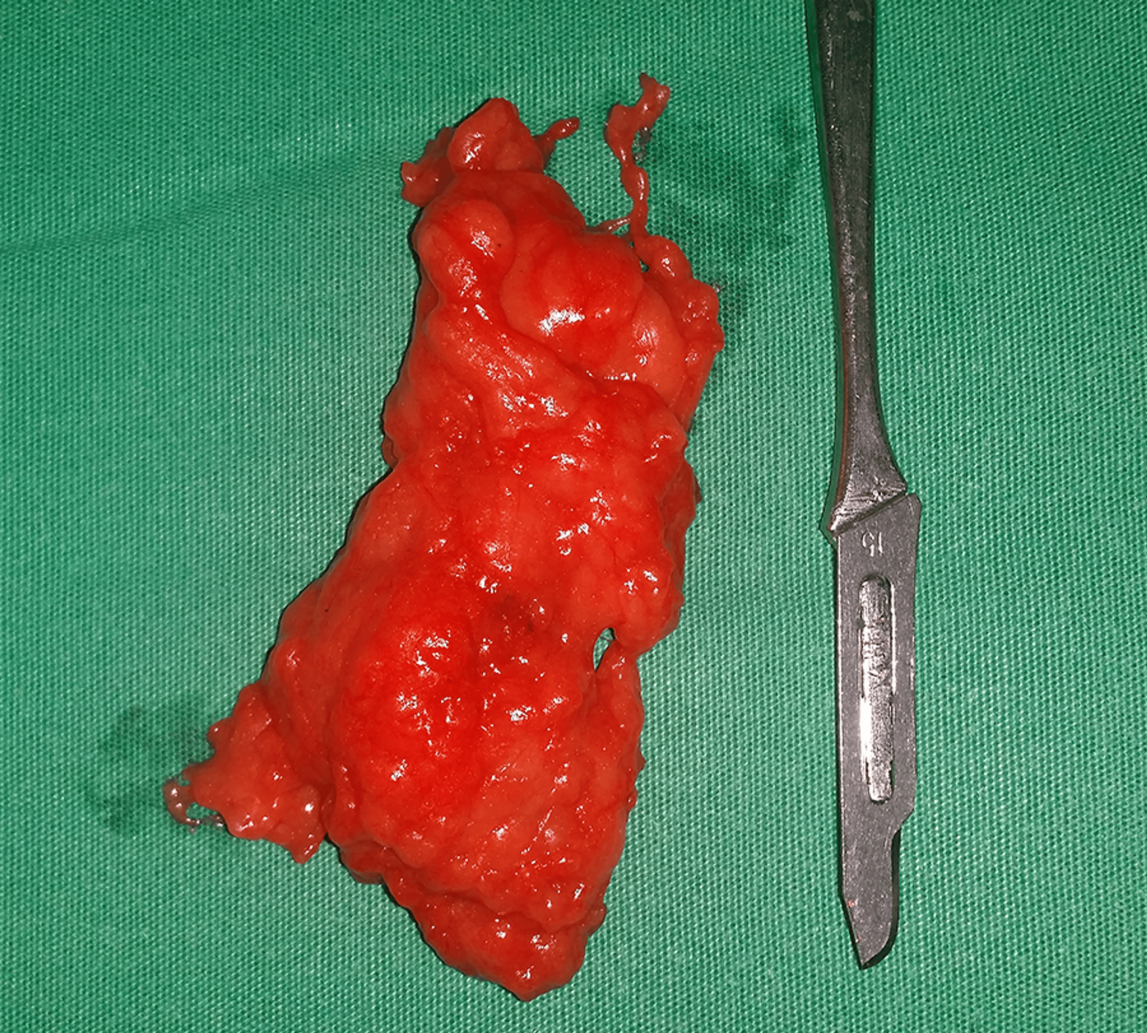
Macroscopic appearance of the excised tumor

The macroscopic examination of the resected tumor revealed that the tumor measured 7 cm × 5.3 cm × 1.6 cm and was separated from the surrounding tissues by a thin capsule (Figure 5).

**Figure 6:**
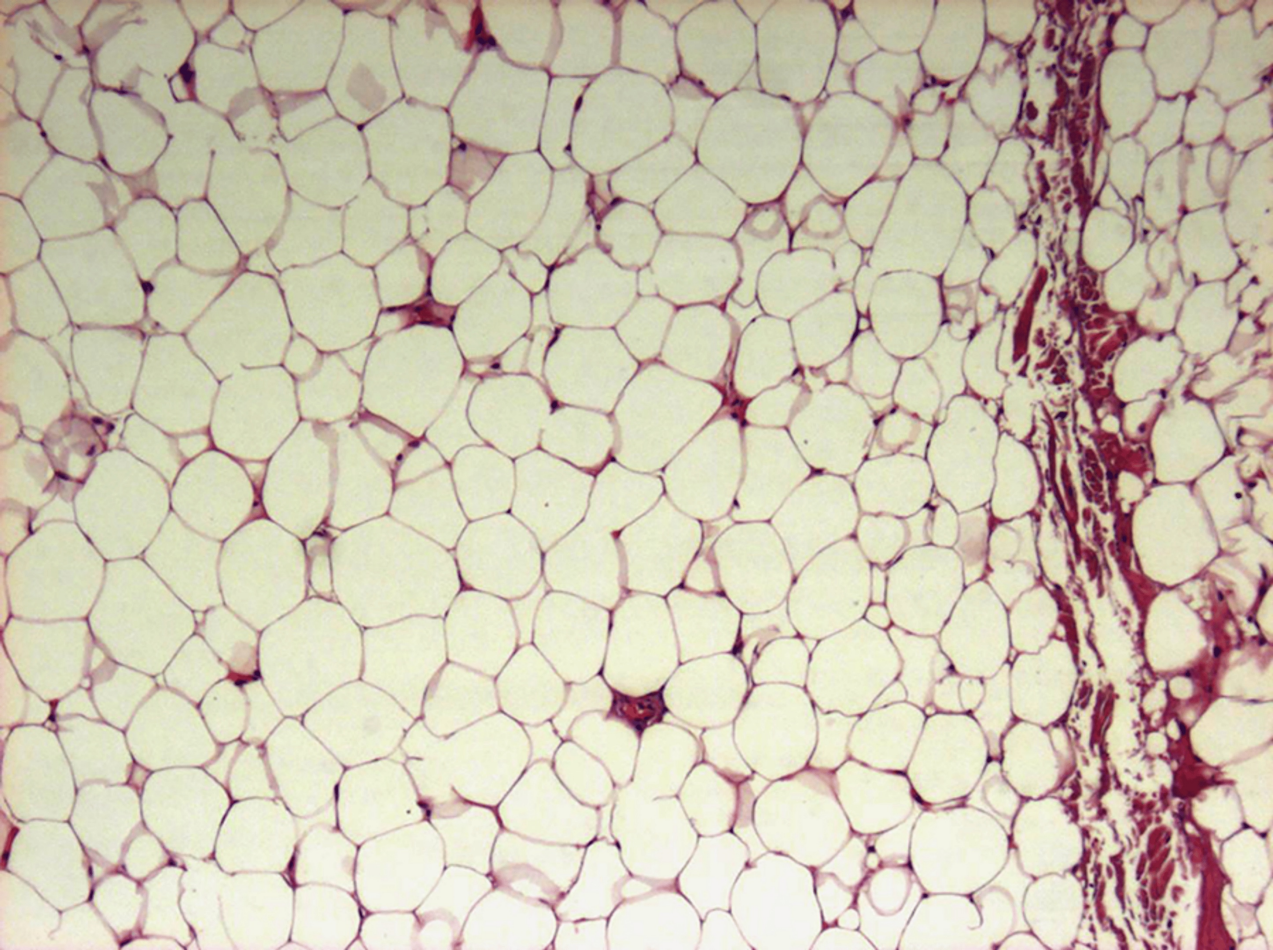
Photomicrograph demonstrating aggregates of mature adipocytes (H&E stain, 100×)

Histopathologic examination showed that the tumor was composed of mature adipocytes without signs of atypia; a definitive diagnosis of lipoma was made (Figure 6).

The patient was hospitalized and monitored for two days and then discharged in good general condition with instructions and under antibiotic treatment. Two weeks after surgical excision, the patient developed an abscess that was attributed to poor oral hygiene and was treated with incision and drainage and administration of antibiotics. The patient is under follow-up without any signs of recurrence.

## Discussion

Lipomas are relatively uncommon in the oral and maxillofacial region, despite the fact that benign lipomas are the most common mesenchymal tumors of the soft tissues [5-8].

Oral lipoma is usually present in adults 50-70 years old, although it may occur at any age, with a slight predilection for women [9,10]. The predilection for men reported in the study of Furlong et al. [7] could be explained by the fact that the study was conducted in soldiers’ populations in the USA. The etiopathogenesis of lipomas is not known, and no systemic factors have been implicated, although a correlation with obesity has been reported (similar to our case) [11].

On clinical examination, oral lipomas usually present as sessile or, less commonly, pedunculated nodules covered by yellowish to normal mucosa with an intact surface [7,9,10]. Various entities presenting as oral masses or swelling may be entertained in the differential diagnosis, including reactive growths, e.g., fibroma; other benign mesenchymal neoplasms, such as neurofibroma or leiomyoma; cysts, such as oral lymphoepithelial cysts and oral epidermoid cysts; and salivary gland tumors, e.g., pleomorphic adenoma [8-10]. Techniques such as ultrasound examination and MRI can be used to differentiate lipomas from other soft tissue lesions. Ultrasound examination can be used as the initial study. Lipomas are hypoechoic relative to the adjacent muscle and, in most cases, have well-defined borders [12]. In MRI, as the signal intensity of lipomas is similar to subcutaneous fat, the demonstrated suppression sequence can distinguish lipomas from other types of tumors, while fine needle aspiration and cytology examination should be performed to exclude malignancy and confirm the diagnosis [13].

Most tumors are asymptomatic, and the patients may arrive for consultation only if the tumors grow to a considerable size [8]. Lipomas have slow growth with a mean time from first notice to evaluation of three to four years, ranging from a week to three to four decades [10]. In our case, the patient reported that he first noticed the area's swelling four years ago.

The buccal mucosa seems to be the most common site of oral lipomas, accounting for approximately half of the cases, which may be related to the higher abundance of fat in that area [7,8]. However, lipoma of the buccal space is a very rare neoplasm, with only a few cases reported in the literature [14]. Moreover, the size of the lipoma presented in our case is quite impressive, taking into consideration that the mean tumor size of lipomas in the oral and maxillofacial regions is 2.2 cm [7,8]. Large-size oral lipomas have been seldom reported; for example, a lipoma of the buccal mucosa measuring 7.5 cm × 3.0 cm and covered by normal mucosa was reported by Omisakin and Ajike [15] in a 55-year-old female.

Histopathologic examination remains the gold standard for definitive diagnosis [9,10]. The microscopic diagnosis of conventional lipoma, appearing as a well-circumscribed tumor composed of mature adipocytes, is straightforward; however, several variants of lipoma have been described, including fibrolipoma and more rare types, such as spindle cell, pleomorphic and intramuscular lipoma, angiolipoma and sialolipoma [9,10]. In addition, liposarcomas may rarely occur in the oral cavity, presenting as a slow-growing mass with a predilection for the buccal mucosa; again, different subtypes of malignant adipose tissue tumors have been described, including atypical lipomatous tumors (previously known as well-differentiated liposarcoma), myxoid, round cell, pleomorphic and dedifferentiated liposarcoma [16,17]. The former is the most common variant and is a particular consideration in the differential diagnosis given its microscopic similarity to lipoma; the presence of atypical stromal cells and lipoblasts points towards malignancy, while, in equivocal cases, immunohistochemistry reveals MDM2 and CDK4 positivity, and molecular analysis by FISH demonstrates MDM2 gene amplification in atypical lipomatous tumors [17,18].

Regarding the surgical approach used for the excision of the lesion, the intraoral approach that was used in our case has the obvious advantage of the absence of a skin scar on the patient’s face, compared to an extraoral approach, but it is also technically demanding as the buccal space is lateral to the buccinator muscle and underneath the superficial musculoaponeurotic system; care should be taken to avoid damage to the facial nerve or the Stenson duct [19].

## Conclusions

In conclusion, although lipomas represent the most common mesenchymal neoplasm of the soft tissues, they are relatively uncommon in the oral and maxillofacial regions, especially in the buccal space. However, the buccal mucosa seems to be the most common site of oral lipomas, which may be related to the higher abundance of fat in that area. Lipomas of the buccal space rarely reach a particularly large size, and most of them are asymptomatic; thus, patients may seek consultation only if the tumor grows to a considerable size. The intraoral approach for removal of a large buccal space lipoma is preferable from an esthetic standpoint, as no skin scar is present in the patient’s face, but it is equally technically demanding.
